# 贫血对晚期非小细胞肺癌患者化疗疗效及预后的影响

**DOI:** 10.3779/j.issn.1009-3419.2010.10.07

**Published:** 2010-10-20

**Authors:** 崇安 许, 艳 高, 琳 李, 丽丽 邢, 殊 刘

**Affiliations:** 110032 沈阳，中国医科大学附属第四医院肿瘤内科 Department of Oncology Medicine, the Fourth Affiliated Hospital of China Medical University, Shenyang 110032, China

**Keywords:** 肺肿瘤, 癌性贫血, 化疗疗效, 预后, 生活质量, Lung neoplasms, Cancer-related anemia, Chemotherapy efficacy, Prognosis, Quality of life

## Abstract

**背景与目的:**

癌性贫血是非小细胞肺癌（non-small cell lung cancer, NSCLC）的常见并发症，不但严重影响NSCLC患者的生活质量（quality of life, QOL），还影响其化疗疗效和预后，本研究通过统计NSCLC患者化疗前后贫血发生率，分析其危险因素，探讨贫血对NSCLC患者QOL及化疗疗效和预后的影响。

**方法:**

回顾性分析我院2007年1月-2008年12月期间住院的140例NSCLC患者化疗前后血红蛋白水平变化，探讨发生贫血的危险因素，分析其与化疗疗效、预后的关系；并应用QOL量表EORTCQLQ-C30中文版评价贫血对NSCLC患者QOL的影响。

**结果:**

140例NSCLC患者，化疗2周期后贫血发生率明显高于化疗前（71.4% *vs* 47.1%, *P* < 0.001），贫血严重程度随着化疗周期的增加而加重。单因素和多因素*Logistic*回归分析显示，年龄、临床分期、PS评分、白蛋白水平与治疗前发生癌性贫血密切相关；而多因素*Logistic*分析显示，仅白蛋白水平是引发治疗后贫血的危险因素。贫血者与非贫血者的QOL量表得分在症状、躯体功能及整体生活质量均有显著差异（*P* < 0.05）。无论化疗前发生的贫血还是化疗导致的贫血均显著降低NSCLC患者化疗的疗效。癌性贫血严重影响患者的预后，发生癌性贫血者的中位生存期明显短于无贫血者（7个月*vs* 13个月，*P* < 0.001）；化疗前就发生癌性贫血者的中位生存期亦明显短于化疗前无贫血者（7个月*vs* 11个月，*P* < 0.001）。*Cox*多因素回归分析证实，贫血、临床分期、PS评分、白蛋白水平是影响NSCLC患者预后的独立因素。

**结论:**

NSCLC患者有较高的贫血尤其是化疗相关性贫血的发生率，年龄、临床分期、PS评分、白蛋白水平是引发治疗前癌性贫血的危险因素。贫血不但降低NSCLC患者的QOL、降低化疗疗效，还缩短其生存期，是影响预后的独立因素。

## Abstract (en)

**Background and objective:**

Cancer-related anemia is the common complication of non-small cell lung cancer (NSCLC), which affects the quality of life (QOL), chemotherapy efficacy and prognosis in patients with NSCLC. In this study, we statistically analysed the incidence of anemia in NSCLC patients before and after chemotherapy and related risk factors, and investigated the impact of anemia on QOL, chemotherapy efficacy and prognosis in patients with NSCLC.

**Methods:**

Clinical data of 140 patients with NSCLC from January 2007 to December 2008 were collected and retrospectively analysed for the changes in hemoglobin levels before and after chemotherapy, the risk factors of anemia, and the relationship with chemotherapy efficacy and prognosis. Application of QOL scale (EORTCQLQ-C30, Chinese version) to evaluate the impact of anemia on QOL in patients with advanced NSCLC.

**Results:**

Total 140 cases of NSCLC patients, the incidence of anemia after two cycles of chemotherapy was significantly higher than that before chemotherapy (71.4% *vs* 47.1%, *P* < 0.001), and the severity of anemia increased simultaneously with the cycles of chemotherapy. According to univariete and multivariate *Logstic* regression analysis, age, clinical stage, PS score and albumin levels were closely related to pre-treatment cancer-related anemia. However, multivariate *Logistic* regression analysis confirmed that only albumin levels was the risk factors of anemia. QOL scores on physical function, symptoms and overall quality of life were significantly different in anemia and nonanemic patients (*P* < 0.05). Anemia occurred before and after chemotherapy were significantly lower chemotherapy efficacy in patients with advanced NSCLC. Cancer-related anemia obviously impacted on the prognosis of patients, the median survival of cancer-related anemia patients was significantly shorter than those without anemia (7 months *vs* 13 months, *P* < 0.001), and the median survival of cancer-related anemia patients before chemotherapy was significantly longer than those with anemia (7 months *vs* 11 months, *P* < 0.001). *Cox* multivariate regression analysis confirmed that anemia, clinical stage, PS score, albumin level were the independent prognostic factors in advanced NSCLC.

**Conclusions:**

NSCLC patients had a higher incidence of anemia, especially the incidence of chemotherapy-related anemia, age, clinical stage, PS score, albumin levels were the risk factors of pre-treatment cancer-related anemia. NSCLC patients with anemia had lower QOL and chemotherapy efficacy, and shorter lifetime. Anemia was an independent prognostic factor in NSCLC patients.

癌性贫血又称肿瘤相关性贫血是由于恶性肿瘤本身和治疗相关导致的贫血，是恶性肿瘤患者常见的并发症，恶性肿瘤患者癌性贫血发生率高达50%以上^[1]^。肺癌是并发癌性贫血较高的恶性肿瘤，约占肺癌80%的非小细胞肺癌（non-small cell lung cancer, NSCLC），由于症状出现晚且缺乏特异性，故确诊时约80%已为晚期，化疗是其主要治疗手段，化疗虽然可改善NSCLC患者的生活质量，延长其生存期^[2]^，但是化疗诱发的化疗相关性贫血却显著降低患者的生活质量（quality of life, QOL），并严重影响患者的治疗效果和预后，甚至危及生命。为探讨NSCLC患者贫血的发生率和危险因素，探究其与NSCLC患者QOL、化疗疗效以及预后的关系，现将我院140例晚期NSCLC患者化疗期间发生贫血的情况报告如下。

## 1 资料和方法

### 1.1 病例资料

#### 1.1.1 病例来源

所有病例均来源于2007年1月-2008年12月中国医科大学附属第四医院收治的住院患者。

#### 1.1.2 入组标准

① 能承受至少2周期一线联合化疗，并且经细胞学或组织病理学确诊的初治NSCLC患者，并有客观病灶可作为疗效评价；②不可切除性Ⅲ期-Ⅳ期NSCLC患者；③所有患者化疗前均未接受放射治疗；④体力状态（performance score, PS）评分为0分-2分；⑤化疗前4周内未输血，且血红蛋白≥80 g/L；⑥心、肝、肾功能和骨髓功能正常，没有严重的合并症；⑦预计生存期≥3个月；⑧签署化疗同意书和病情告知书。

#### 1.1.3 排除标准

① 年龄 > 80岁；②脑转移患者；③并发慢性感染或炎症；④合并其他血液病患者（如再生障碍性贫血）。

#### 1.1.4 病例特征

本组140例经病理组织学或细胞学证实NSCLC患者，男97例，女43例，中位年龄63岁（27岁-80岁）；腺癌86例，鳞癌54例。按国际抗癌联盟（International Union Against Cancer, UICC）肺癌TNM临床分期标准（1997年）Ⅲb期77例、Ⅳ期63例。发生骨转移34例，肝转移5例。PS评分0分45例，1分75例，2分20例。

### 1.2 方法

#### 1.2.1 化疗方案

采用含铂类（顺铂或卡铂）为主的一线两药联合化疗方案，其中，EP（足叶乙甙+铂类）方案37例，NP（铂类+长春瑞滨）方案58例，GP（铂类+吉西他滨）方案7例，TP（铂类+紫杉醇）方案9例，DP（铂类+多西他赛）方案29例。所有病例均在化疗2周期后评定疗效。

#### 1.2.2 血红蛋白（hemoglobin, Hb）测定方法

抽取清晨静脉血2 mL，置于EDTA抗凝管中，2 h内应用血细胞分析仪（SE-2100，Sysmex公司）检测Hb值。

### 1.3 癌性贫血诊断标准

按照1999年WHO标准，Hb < 110 g/L为贫血，根据其严重程度将贫血分为5级：0级（正常）：Hb≥110 g/L；Ⅰ级（轻度）：Hb 95.0 g/L-109.0 g/ L；Ⅱ级（中度）：Hb 80.0 g/L-94.0 g/L；Ⅲ级（重度）：Hb 65.0 g/L -79.0 g/L；Ⅳ级（极重度，危及生命）：Hb < 65.0 g/L。

### 1.4 疗效评价标准

#### 1.4.1 客观疗效

按RECIST（Response Evaluation Criteria in Solid Tumors）标准进行疗效评价，分为完全缓解（complete response, CR）：全部病灶完全消失，持续4周；部分缓解（partial response, PR）：病灶的最大直径与最大垂直径乘积缩小50%以上，持续4周；疾病稳定（stable disease, SD）：病灶的最大直径与最大垂直径乘积缩小不到50%或增大未超过25%，持续至少4周且无新病灶出现；疾病进展（progressive disease, PD）：肿瘤病灶两径乘积增大超过25%，或出现新病灶。化疗2周期评定疗效，CR+PR视为客观有效（objective response, OR），SD+PD视为无效。

#### 1.4.2 生存期计算

生存期从确诊之日始，直至死亡或最后一次随访，随访截止时间为2009年10月。随访方法采用回院治疗、复查、走访、随访信或电话方式随访。

### 1.5 生活质量评价标准

采用含15个子量表（包括5个功能子量表、3个症状子量表、1个总体健康状况/生活质量子量表和6个单一条目），共计30个条目的生活质量量表EORTCQLQ-C30（V3.0）中文版^[3]^，评价140例NSCLC患者QOL，探讨癌性贫血与NSCLC患者QOL的相关性。本研究评价指标主要包括和癌性贫血密切相关的1个症状（疲劳）子量表；1个失眠项目；1个功能（躯体功能）子量表及l个整体生活质量量表。每个子量表的内容称为维度。症状子量表/条目得分越高表示症状越重，功能子量表/条目得分越高表示功能越好，整体生活质量量表得分越高表示整体生活质量越好。生活质量量表EORTCQLQ-C30是癌症患者生命质量测定量表体系中的核心量表，具有较好的信度、效度及可行性和一定反应度。具体评分如下，总体健康状况/生命质量子量表分为7个等级，根据其回答选项，计为1分-7分；其它条目分为4个等级，计1分-4分。为了使得各领域得分能相互比较，采用极差化方法进行线性变换，将粗分转化为在0-100内取值的标准化得分。

### 1.6 统计学方法

采用SPSS 11.5软件进行数据分析，率的比较采用χ^2^检验，两样本间的比较采用*t*检验，采用*Logistic*回归模型计算各变量的比值比及相应的95%CI；纳入回归方程的有年龄、性别、病理类型、临床分期、PS评分、白蛋白水平。采用*Pearson*相关系数评价各变量之间的相关性。生存分析采用*Kaplan-Meier*法，组间比较采用*Log-rank*检验；对影响生存预后的联合效应采用*Cox*比例风险模型进行多因素分析，纳入的自变量为年龄、性别、临床分期、化疗方案、PS评分、贫血、白蛋白水平，因变量为生存期；以*P* < 0.05为有统计学差异，所有检验和*P*值均为双侧。

## 2 结果

### 2.1 贫血发生率及化疗前后Hb水平

#### 2.1.1 贫血发生率及化疗前后Hb水平

本组140例NSCLC患者中，化疗前贫血发生率为47.1%（66/140），化疗2周期后贫血发生率为71.4%（100/140），明显高于化疗前（χ^2^=17.104, *P* < 0.001）化疗4周期后贫血发生率为78.6%（110/140），虽然高于化疗2周期，但无统计学意义（*P*=0.168）。化疗前无贫血的74例患者中，化疗4周期后44例发生贫血，化疗相关性贫血发生率为59.5%。根据化疗前后有无贫血将患者分为3组，A组（化疗前后均无贫血）、B组（化疗前无贫血化疗后有贫血）和C组（化疗前后均有贫血）；三组化疗前后Hb水平变化见表 1。

**1:** 化疗对NSCLC患者血红蛋白的影响（g/L, Mean±SD） The impact of chemotherapy on hemoglobin in NSCLC patients (g/L, Mean±SD)

Group	*n*	Before chemotherapy	After 2 cycles of chemotherapy	After 4 cycles of chemotherapy
A	30	139.4±10.36	128.4±15.30Δ	120.3±13.02^**^
B	44	127.8±8.55	92.8±11.69^*^	86.5±14.71^**^
C	66	99.4±7.13	84.2±8.79^*^	79.5±8.97^**^
Group A: Before and after chemotherapy without anemia; Group B: Before chemotherapy without anemia and after chemotherapy with anemia; Group C: Before and after chemotherapy with anemia; Δ: The self-comparative between before chemotherapy and after 2 cycles of chemotherapy, *P* < 0.005; ^*^: The self-comparative between before chemotherapy and after 2 cycles of chemotherapy, *P* < 0.001; ^**^: The self-comparative between after 2 and 4 cycles of chemotherapy, *P* < 0.001.				

#### 2.1.2 癌性贫血的性质

100例癌性贫血的NSCLC患者中，绝大多数（85%）为与癌症相关的慢性贫血，其中，血清铁（正常值：9 μmol/L -30 μmol/L）正常68例（68%），升高19例（19%），降低13例（13%）；铁蛋白（正常值：男性30 ng/L-400 ng/mL，女性13 ng/L-150 ng/mL）正常57例（57%），升高34例（34%），降低9例（9 %）；确诊缺铁性贫血8例（8 %）。7 5例行叶酸和维生素B_12_检测，其中叶酸（正常值4.6 ng/L -18.7 ng/mL）正常68例（90.7%），升高3例（4.0%），降低4例（5.3%）；维生素B_12_（正常值240 ng/L-900 pg/mL）正常59例（78.7%），升高13例（17.3%），降低3例（4.0%），确诊营养性贫血7例（7%）。

### 2.2 癌性贫血的危险因素

治疗前癌性贫血的单因素分析显示，治疗前NSCLC患者的癌性贫血与年龄、临床分期、PS评分、白蛋白水平密切相关（表 2）。经多因素*Logistic*回归分析证实，年龄、临床分期、PS评分、白蛋白水平是引发癌性贫血的危险因素，*P*值分别为0.001、0.001、0.007、 < 0.001。63例Ⅳ期患者中，并发骨转移的45例（71.4%）患者中，有33例（73.3%）并发癌性贫血，未发生骨转移的18例患者中，有8例（44.4%）发生癌性贫血，二者有明显的统计学差异（χ^2^=5.659, *P*=0.017）。

**2:** 癌性贫血危险因素的单因素分析 Univariate analysis of risk factors for anemia

Risk factors	*n*	Number of anemia	Incidence of anemia	*χ*^2^	*P*
Gender				0.072	0.789
Male	97	45	46.4%		
Female	43	21	48.8%		
Age (year)				6.966	0.008
< 65	80	30	37.5%		
≥65	60	36	60.0%		
Stage				14.789	< 0.001
Ⅲ	77	25	32.5%		
Ⅳ	63	41	65.1%		
PS scores				13.420	< 0.001
0-1	120	49	40.8%		
2	20	17	85.0%		
Serum albumin (g/L)				20.813	< 0.001
≤30	69	46	66.7%		
> 30	71	20	28.2%		
Histological type				2.497	0.114
Adenocarcinoma	86	36	41.9%		
Squamous cell carcinoma	54	30	55.6%		

治疗后癌性贫血的单因素分析显示，治疗后NSCLC患者的癌性贫血与临床分期、PS评分、白蛋白水平密切相关，*P*值分别为0.007、0.012、 < 0.001。但经多因素*Logistic*回归分析证实，仅白蛋白水平是引发治疗后癌性贫血的危险因素（*P*=0.001）。

### 2.3 贫血对化疗疗效的影响

#### 2.3.1 总体疗效

所有研究对象平均接受4个周期（范围2个周期-8个周期）的化疗。140例NSCLC患者化疗2个疗程后53例（37.9%）达到OR（CR+PR），其中CR 3例（2.2%），PR 50例（35.7%），SD 50例（35.7%），PD 37例（26.4%）。

#### 2.3.2 贫血对化疗疗效的影响

无论化疗前发生贫血，还是化疗导致的贫血均显著降低NSCLC患者的化疗疗效（表 3）。

**3:** 贫血对NSCLC患者化疗疗效的影响 The impact of anemia on chemotherapy efficacy of NSCLC patients

Influencing factors	*n*	OR	*χ*^2^	*P*
Pre-chemotherapy			12.219	< 0.001
Anemia	74	24.3% (18/74)		
Normal	66	53.0% (35/66)		
Post-chemotherapy			11.671	0.001
Anemia	100	29.0% (29/100)		
Normal	40	60.0% (24/40)		
OR: objective response.				

### 2.4 贫血对预后的影响

在研究期间，140例患者死亡115例，所有患者的中位生存期为8.5个月。无癌性贫血者的中位生存期明显长于有癌性贫血者（13个月*vs*7个月，*P* < 0.001）；化疗前后均无贫血者的中位生存期明显长于化疗前无贫血而化疗后贫血者（13个月*vs* 9个月，*P* < 0.001）；化疗前无贫血而化疗后贫血者的中位生存期明显长于化疗前后均贫血者（9个月*vs* 7个月，*P* < 0.001）；化疗前无贫血者中位生存期明显长于化疗前贫血者（11个月*vs* 7个月，*P* < 0.001）（图 1）。

**1:**
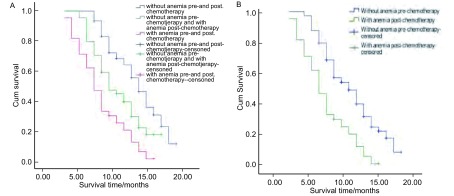
NSCLC患者是否并发癌性贫血的生存曲线。A：化疗后；B：化疗前。 The Kaplan-Meier survival curve of NSCLC patients. A: post-chemotherapy; B: pre-chemotherapy.

### 2.5 影响预后的危险因素

单因素分析结果显示，临床分期、PS评分、白蛋白水平、癌性贫血与NSCLC患者预后生存期密切相关（表 4）。

**4:** 影响NSCLC患者预后的单因素分析 Univariate survival analysis of NSCLC patients

Risk factors	*n*	Median survival time (months)	95%CI	*P*
Gender				0.364 5
Male	97	9	8.15-9.85	
Female	43	10	6.85-13.15	
Age (year)				0.497 4
> 65	80	8	6.98-9.02	
≥65	60	10	8.3-11.7	
Stage				0.001 9
Ⅲ	77	11	9.35-12.65	
Ⅳ	63	8	7.22-8.78	
PS scores				< 0.000 1
0-1	120	10	8.42-11.58	
2	20	6	4.55-7.45	
Serum albumin (g/L)				0.001 3
≤30	69	7	6.1-7.9	
> 30	71	11	9.46-12.54	
Cancer-related anemia				0.000 1
Yes	110	8	7.27-8.73	
No	30	13	10.98-15.02	
Chemotherapy regimen				0.211 7
Paclitaxel	38	9	5.68-12.32	
Non-paclitaxel	102	9	8.11-9.89	

*Cox*回归模型分析显示，癌性贫血（R R = 1.956, 95%CI: 1.125-3.402, *P*=0.017）、临床分期（RR=1.477, 95%CI: 1.003-2.174, *P*=0.048）、PS评分（RR=2.895, 95%CI: 1.714-4.889, *P* < 0.001）、白蛋白水平（RR=1.555, 95%CI: 1.063-2.277, *P*=0.023）是影响NSCLC患者预后的独立因素。

### 2.6 癌性贫血对生活质量的影响

应用生活质量量表EORTC QLQ - C30（V3.0）中文版，评价癌性贫血对NSCLC患者生活质量的影响，结果显示，贫血者与非贫血者的EORTC QLQ-C30得分在躯体功能、症状及整体生活质量方面均有统计学差异（*P* < 0.05）。贫血患者症状评分较无贫血患者高，分别为：疲劳状况评分高16分（*P*=0.016），失眠状况评分高23分（*P*=0.001）；非贫血者比贫血者的躯体功能评分高15分（*P*=0.009）、整体生活质量提高24分（*P* < 0.001）。

应用直线相关方程计算结果显示，贫血与生活质量各维度之间存在直线相关性。贫血与躯体功能（*P*=0.005, *r*=-0.238）和整体生活质量（*P* < 0.001, *r*=-0.336）呈明显负相关，与疲劳（*P*=0.001, *r*=0.269）及失眠（*P* < 0.001, *r*=0.310）呈明显正相关。

## 3 讨论

贫血是多种疾病常见的并发症，癌性贫血是恶性肿瘤，尤其晚期肿瘤患者最常见的并发症之一。欧洲癌症贫血调查组对15 367例肿瘤患者调查发现，癌性贫血发生率均多超过50%，其中肺癌患者贫血发生率为77%^[4]^，国内陆崇等^[5]^报道的中晚期NSCLC患者化疗后贫血的发生率为76.2%。Dalton等^[6]^调查了28个肿瘤中心接受化疗的2 821例肿瘤患者，发现癌性贫血的发生率由化疗后第1周期的17.0%上升至第6周期的35.0%（其中肺癌为51.0%）。本组140例NSCLC患者中，化疗前贫血发生率为47.1%，化疗2周期后贫血发生率为71.4%，化疗4周期后贫血发生率为78.6%，化疗前无贫血的74例患者，化疗后化疗相关性贫血发生率为59.9%。而且化疗后Hb值明显低于治疗前，支持在疾病的发展过程中由于化疗以及疾病本身等众多原因均可诱发或加重癌性贫血^[7, 8]^，且随着化疗周期的增加，不但增加贫血的发生率，而且加重贫血的程度^[6]^。

除化疗及NSCLC疾病本身等原因诱发贫血外，本研究还发现年龄、临床分期、PS评分、白蛋白水平与治疗前NSCLC患者发生贫血密切相关，经多因素*Logistic*回归分析证实，临床分期、PS评分、白蛋白水平是诱发治疗前NSCLC患者贫血的危险因素。单因素分析发现，治疗后发生癌性贫血与临床分期、PS评分、白蛋白水平密切相关，但多因素回归分析显示，仅白蛋白水平是引发化疗后癌性贫血的危险因素。

由于贫血可导致肿瘤组织缺氧^[9]^，而缺氧可使肿瘤细胞的基因表达改变，进而引起蛋白质组和基因组发生改变，从而使得肿瘤的侵袭性增强，导致肿瘤进展^[10]^。另外，缺氧可促进肿瘤新生血管形成，并可诱导多种促凝因子表达，使肿瘤细胞更易于浸润和转移^[11]^。缺氧不但促使肿瘤进展和转移而且可使肿瘤对放化疗的抵抗力增加，从而降低治疗效果，影响预后^[12]^。本组结果显示，化疗前无贫血的NSCLC患者化疗后OR明显高于有贫血者（53.0% *vs* 24.3%, *P* < 0.001），化疗后未发生贫血患者的OR亦明显高于化疗后伴贫血者（60.0% *vs* 29.0%, *P*=0.001）。单因素分析显示，贫血、临床分期、PS评分、白蛋白水平与NSCLC患者预后密切相关。无癌性贫血者的中位生存期明显长于有癌性贫血者（13个月*vs* 7个月，*P* < 0.001）；化疗前后均无贫血者的中位生存期明显长于化疗前无贫血而化疗后贫血者（13个月*vs* 9个月，*P* < 0.001）；化疗前无贫血而化疗后贫血者的中位生存期明显长于化疗前后均贫血者（9个月*vs* 7个月，*P* < 0.001）；化疗前无贫血者中位生存期明显长于化疗前贫血者（11个月*vs* 7个月，*P* < 0.001）。*Cox*回归模型分析也进一步证实了癌性贫血、临床分期、PS评分、白蛋白水平是影响NSCLC患者预后的独立因素。上述结果表明，癌性贫血不但降低NSCLC患者化疗疗效，缩短其生存期，还是影响其预后的独立因素^[13]^。

由于贫血影响患者的组织供氧，影响脏器功能，常导致患者的情绪低落，认知功能下降，乏力，心动过速，心、肺、肾功能不全等，主要表现为乏力、嗜睡、抑郁、呼吸困难等症状，直接影响患者的QOL^[14, 15]^。本研究结果同样显示，贫血显著影响NSCLC患者的QOL，贫血者与非贫血者的EORTC QLQ-C30得分在躯体功能、症状及整体QOL方面均有显著差异（*P* < 0.05）。贫血与QOL各维度之间存在直线相关性。贫血与躯体功能（*P*=0.005）和整体生活质量（*P* < 0.001）呈明显负相关，与疲劳（*P*=0.001）及失眠（*P* < 0.001）呈明显正相关。

晚期NSCLC目前仍是不可治愈性疾病，其治疗主要目的是提高QOL和延长生存，而贫血不但影响治疗效果和生存，还影响患者的QOL，因此，治疗癌性贫血无论对患者本身，还是对NSCLC治疗都是有益的。

综上所述，由于诱发NSCLC患者贫血的原因众多，尤其是化疗导致的化疗相关性贫血发生率很高，因贫血严重影响患者的QOL，降低治疗效果，缩短其生存期，应引起我们足够的重视。对于以改善症状，提高QOL，延长生存为主要目的的不可治愈的晚期NSCLC患者来讲，纠正癌性贫血不但可以提高化疗疗效，延长生存期，还能提高患者的QOL。因此，对于治疗前发生的贫血，应积极寻找原因，针对病因治疗，在积极治疗原发病的同时给予支持、对症治疗；对于化疗相关性贫血，应在给予EPO的同时给予支持、对症治疗，并采取积极的预防措施，以减少癌性贫血的发生，从而提高NSCLC患者的治疗效果，改善生活质量，延长生存期。

## References

[1] Mercadante S, Gebbia V, Marrazzo A (2000). Anaemia in cancer: pathophysiology and treatment. Cancer Treat Rev.

[2] Pfister DG, Johnson DH, Azzoli CG (2004). A American Society of Clinical Oncology treatment of unresectable non-small-cell lung cancer guideline: update 2003. J Clin Oncol.

[3] Wang CH, Chen QM, Zhang CZ (2005). Evaluate the quality of life in cancer patents with EORT QLQC-30 Chinese version. J Prac Oncol.

[4] Ludwig H, Van Belle S, Barrett-Lee P (2004). The European Cancer Anaemia Survey (ECAS): a large, multinational, prospective survey defining the prevalence, incidence, and treatment of anaemia in cancer patients. Eur J Cancer.

[5] Lu C, Liu KF, Zhang KN (2007). Analysis the clinical features of chemotherapy-related anemia in advanced non-small cell lung cancer. J Prac Med.

[6] Dalton JD, Bailey NP, Barrent-Lee PJ (1998). Multicenter UK audit of anemia in patients receiving cytotoxic chemotherapy. Proc ASCO.

[7] Barrett-Lee PJ, Bailey NP, O'Brien ME (2000). Large-scale UK audit of blood transfusion requirements and anaemia in patients receiving cytotoxic chemotherapy. Br J Cancer.

[8] Campos S (2002). The impact of anemia and its treatment on patients with gynecologic malignancies. Semin Oncol.

[9] Blackwell K, Gascón P, Sigounas G (2004). rHuEPO and improved treatment outcomes: potential modes of action. Oncologist.

[10] Vaupel P, Mayer A, Briest S (2003). Oxygenation gain factor: a novel parameter characterizing the association between hemoglobin level and the oxygenation status of breast cancers. Cancer Res.

[11] Denko NC, Giaccia AJ (2001). Tumor hypoxia, the physiological link between Trousseau's syndrome (carcinoma-induced coagulopathy) and metastasis. Cancer Res.

[12] Semenza GL (2002). HIF-1 and tumor progression: pathophysiology and therapeutics. Trends Mol Med.

[13] Caro JJ, Salas M, Ward A (2001). Anemia as an independent prognostic factor for survival in patients with cancer: a systemic, quantitative review. Cancer.

[14] Cella D (1998). Factors influencing quality of life in cancer patients: anemia and fatigue. Semin Onocol.

[15] Borget I, Tilleul P, Baud M (2007). A prospective study of quality of life and treatment of chemotherapy-induced anemia in lung cancer. Rev Mal Respir.

